# The influence of the casting process on the internal structure and physical properties of hemp-lime

**DOI:** 10.1617/s11527-016-0976-4

**Published:** 2016-12-09

**Authors:** Joseph Williams, Mike Lawrence, Pete Walker

**Affiliations:** BRE Centre for Innovative Construction Materials, Department of Architecture and Civil Engineering, University of Bath, Bath, BA2 7AY UK

**Keywords:** Hemp-lime, Bio-aggregate, Image analysis, Compressive strength, Thermal conductivity, Directional modeling

## Abstract

Bio-aggregate composites such as hemp-lime offer a more sustainable alternative to traditional walling infill material. Hemp-lime, whether in situ or prefabricated, is generally either cast or sprayed, which results in a directionally dependent, typically layered, physical structure. This paper considers the impact of compaction and layering on the directional thermal conductivity, compressive strength and internal structure of the material through use of a novel image analysis method. The results presented indicate that production variables have a significant, and crucially, directionally dependent impact on the thermal and mechanical properties of cast hemp-lime.

## Introduction

Hemp-lime is a bio-aggregate composite material produced by combining the chopped woody core of the hemp plant (shiv), a lime-based binder and water. The resulting mixture can be cast or sprayed into place and cures to form a rigid but highly voided structure. The large proportion of bio-sourced material means that hemp-lime is a net absorber of carbon dioxide that is essentially sustainable to produce [1–3]. The multi scaled porous structure within hemp-lime produces a high thermal resistivity alongside a high thermal storage capacity and moisture buffering properties, making it a desirable infill insulation material for use in building envelopes [4–7].

The most common method used to manufacture hemp-lime is to mix the components into a wet mix that is cast within formwork or block moulds. The process of casting is usually conducted in layers that are individually placed by tamping. The depth of layers, the degree of compaction and the direction of compaction with respect to the direction of thermal and mechanical loading are all variables within this process. The distribution of density within cast material is known to be dependent on depth within a single layer and globally a function of compaction [8, 9]; it is also known that the casting process produces an orientated internal structure [10]. Taken together these variables will in part determine the internal topology of the material and thus have an influence on the physical properties.

The effect of compaction of hemp-lime on the mechanical and thermal properties has been studied previously. Nguyen et al. [8, 9] found that static load compaction of fresh hemp-lime during casting can improve the peak compressive strength and stiffness of the material; the thermal conductivity was also seen to increase but to a lesser extent. The results are attributed to a reduction of inter particle voids that would reduce the volume fraction of thermally resistive air and increase the volume fraction of structural binder. It is proposed elsewhere [11–16] and widely accepted that compressive strength and thermal conductivity of hemp-lime follows an approximate linear relation to density for this reason.

The density distribution through specimens of sprayed hemp-lime has been assessed previously and found to vary with depth [15]. In addition it has been observed elsewhere that cylinders of hemp lime cast in three layers will tend to fail in compression in the top third [17], suggesting of a lower density of material in this area. It is known from the study of other aggregate material that self-weight and boundary friction will impact density profile of a compacted layer. As these are both a result of the layer size then it is appropriate that for cast material, the layer size may influence the density distribution of the composite and impact on the physical properties. To the authors’ knowledge, no preceding study exists that directly assesses how the size of layers used to cast hemp-lime influences the physical properties.

The aggregate used for hemp-lime comes from the stem of the plant and is therefore generally of an elongated form. The application of any compaction has been shown to produce a preferential orientation of the particles towards stratified planes perpendicular to the compacting force [10] that is widely considered to produce observed anisotropic behaviour [8, 18–21]. The compressive strength, compressive stiffness and failure mode of compacted hemp lime have been shown to depend on whether testing is conducted parallel or perpendicular to the compaction force [21] as has thermal conductivity [8, 20].

This paper considers how the variables of layer size and compaction affect the directional compressive behaviour and thermal conductivity of hemp-lime. Results are presented from uniaxial heat flow test and uniaxial compressive test for five permutations of cast material in two orientations (perpendicular and parallel with respect to casting). Additional information about the internal structure, obtained using a recently developed method of image analysis, is also presented.

## Methodology

### Specimen production

**Fig. 1:**
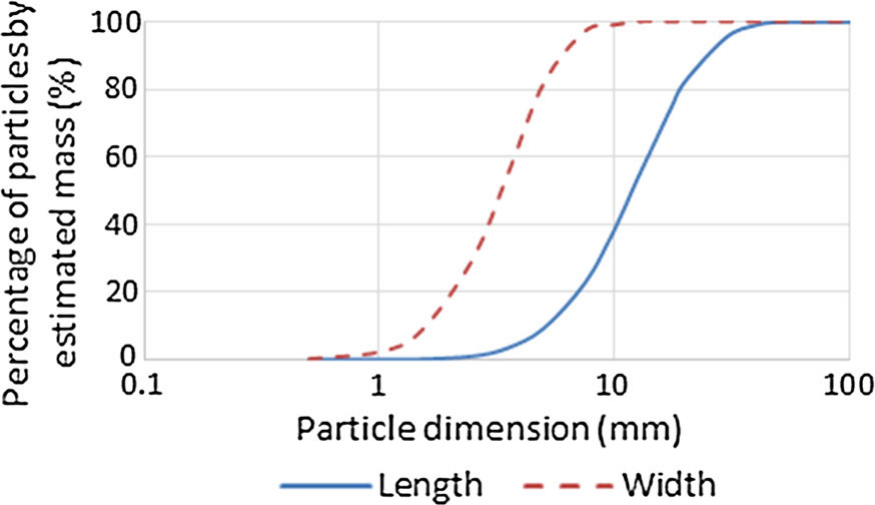
Particle size distribution, length and width, from 2D image analysis

A single mixture of hemp-lime was used to investigate the influence of the internal structure on its properties, with the method of specimen casting the primary variable. The hemp shiv used was produced in France and the bulk properties and particle size distribution are reported in Table 1 and Fig. 1 respectively. The binder used was a commercially available formulated binder for use with hemp, containing mainly a high surface area natural hydraulic lime with the addition of pozzolanic additives. The ratio of binder, hemp and water used throughout the study was 2.25:1.0:3.0 (by mass), which is representative of the ratios widely used for walling applications [22, 23].

**Table 1:** Hemp shiv properties

Country of origin	Bulk density (kg m^−3^)	Mean particle length (mm)	Length standard deviation (mm)	Mean particle width (mm)	Width standard deviation (mm)
France	122	3.34	3.51	1.06	0.987

Mixing of the constituent materials was carried out in a revolving pan mixer. The binder and water were first combined to form a uniform slurry prior to the addition of the hemp shiv. Subsequent mixing was then conducted until a uniform mixture was achieved, paused occasionally to break up any clumps forming by hand. Pre-determined quantities of mixture were weighed out into oiled moulds and tamped to height successively to build up each specimen in the required number of layers. The specimens were left uncovered and transferred to a conditioning room at 20 °C and 70% relative humidity with the moulds being removed after six days.

**Fig. 2:**
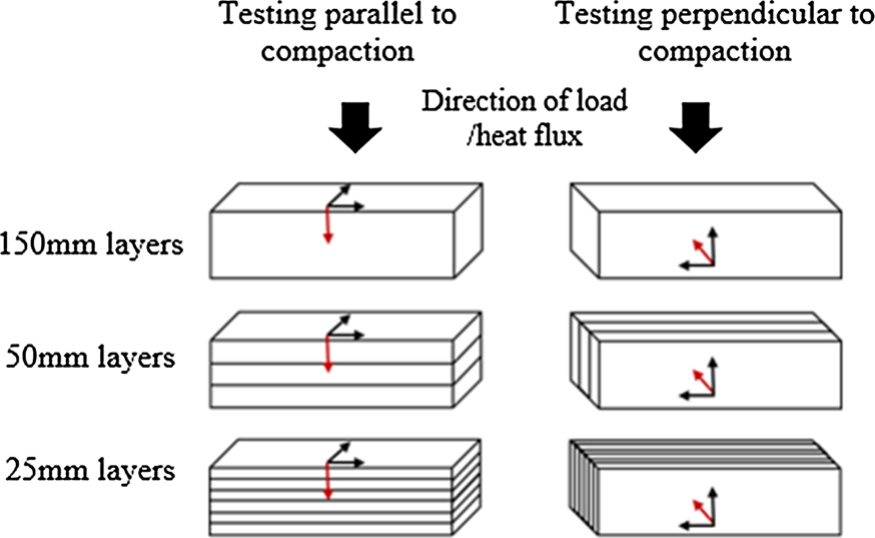
Layering and testing direction arrangements for parallel and perpendicular loading with direction of compaction and preferred plane of orientation of particles indicated by the *red* and *black axis* respectively. (Color figure online)

Specimens were produced using a range of three layer sizes: 25, 50 and 150 mm; and three compaction levels: 30, 45 and 60% volumetric decrease from the uncompact state. Two sets, one to be tested parallel to compaction and one to be tested perpendicular to compaction, were produced for each variant of layer size and compaction level; the full range of permutations considered is detailed in Table 2 and diagrammatically explained in Fig. 2. For each of these permutations, three 150 mm cube specimens were produced for compressive testing with a further three produced for assessment of the internal structure. In addition a single 400 mm by 400 mm by 50 mm specimen was produced for thermal conductivity testing for all permutations except variant 6 where a 150 mm layer would exceed the required 50 mm specimen thickness.

**Table 2:** The design permutations of hemp-lime specimens produced

Variant number	Direction of testing in relation to casting	Compaction (%vol reduction of uncompact)	Size of layers (mm)	Green density (kg m^−3^)	Ave 28 day density (kg m^−3^)
1	Parallel	45	150	649	423
2	Parallel	30	50	577	374
3	Parallel	45	50	649	412
4	Parallel	60	50	721	462
5	Parallel	45	25	649	406
6	Perpendicular	45	150	649	408
7	Perpendicular	30	50	577	355
8	Perpendicular	45	50	649	399
9	Perpendicular	60	50	721	450
10	Perpendicular	45	25	649	411

### Experimental procedure

The methods and specimen ages from casting were chosen based on combination of the British Standards for compressive strength testing of concrete and insulation products [24, 25], as well as preceding studies on hemp-lime [8, 11, 15, 17]. All specimens were removed from the temperature and humidity controlled curing room and tested at 28 days after casting. Specimen masses and dimensions were measured to obtain 28 day densities as well as geometric parameters required for the calculation of stress and strain. Compressive tests were conducted using an Instron 50 KN testing rig at a controlled displacement of 3 mm/min; the inbuilt instrumentation was used to both record load and platen displacement at a resolution of one data point per 0.1 s.

Thermal conductivity measurements were conducted on the 50 mm × 400 mm × 400 mm specimens following oven drying for 48 h at 105°. Mass and dimensions were recorded for all specimens to determine density and for use in calculation of thermal conductivity. The thermal conductivity tests were undertaken using a Fox 600 heat flow meter with a temperature gradient of 10 to 30 °C. Prior to testing the specimens were wrapped with a single layer of clear shrink wrap food packing material in order to both protect the instrument and prevent moisture loss from the specimens during testing.

Assessment of each internal specimen structure was conducted using an original method developed by the authors [10]. In brief it comprises cutting six slices from each specimen using a band saw with the cuts made in the perpendicular plane to the direction of thermal and mechanical loading. The cut faces of each slice were then cast within a low viscosity colored resin, used to highlight the voids within the material, and sanded to reveal a section through the material; the prepared surfaces were ‘imaged’ with a flatbed scanner at a resolution of 1200 dpi. To enhance the contrast of the images produced, a red pigment was added to the slaked binder used in the production of the specimens to enable easier computer recognition of the differing parts of the image: voids (blue resin) binder (red) and hemp shiv (natural yellow).

Analysis of the images was carried out using ImageJ primarily using the measure and partial analysis tools. Prior to analysis the images were smoothed using a median filter to remove noise and then a threshold analysis was undertaken to identify a single constituent and binaries the image. Following this the binary image was further cleaned by use of an opening algorithm to help segregate touching particles and finally a partial analysis was conducted to identify and measure the discreet binary objects (shiv particles) within each image. The orientation of each particle within the image plane was assessed by means of an ellipse fitting process, as described in [10] and the results were combined into frequency analysis of the whole population to give an assessment of global orientation within the plane.

## Results

### Density

The 28 day average density of the different variations of material is given in Table 2 while the dry densities obtained after 48 h oven drying at 105 °C are given in Table 3. From table two it is apparent that the density at 28 days varies with orientation of casting as well as compaction but not layer thickness; the parallel compaction direction is found to be on average 2.6% higher than the parallel direction. From the dry densities, Table 3 again the dry densities are found to be higher in the parallel direction however in this case the average increase is only 1.5% and of comparable magnitude to the average variation found between similar specimens of 1.0%. It is suggested therefore that the casting orientation has bearing on the drying hysterics of the material and thus the 28 day density but negligible impact on the dry density of the material, considered to be solely a result of the compaction where the constituents are kept constant.

**Table 3:** Volumetric proportion of air obtained by 2D image analysis of hemp-lime at different compaction levels

Variant number	Direction of testing in relation to casting	Compaction (%vol reduction of uncompact)	Size of layers (mm)	Volume of air (%macroscopic level)	Dry density (kg m^−3^)
1	Parallel	45	150	30.8	345
2	Parallel	30	50	40.7	319
3	Parallel	45	50	32.7	338
4	Parallel	60	50	23.5	379
5	Parallel	45	25	31.6	338
6	Perpendicular	45	150	35.8	336
7	Perpendicular	30	50	41.7	304
8	Perpendicular	45	50	35.6	337
9	Perpendicular	60	50	23.8	372
10	Perpendicular	45	25	31.6	346

### Compressive behaviour

**Fig. 3:**
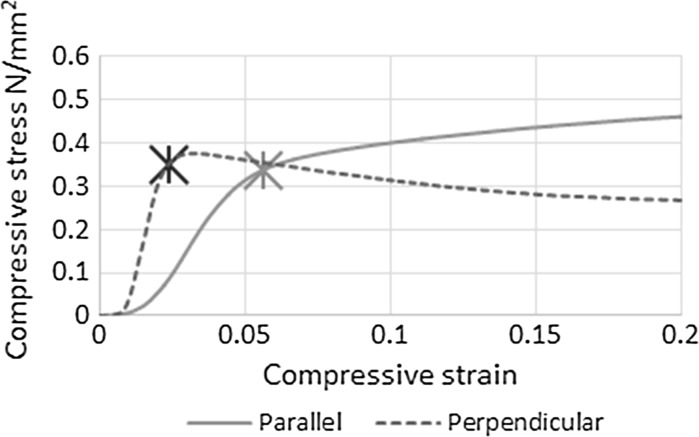
The stress strain relationship for parallel and perpendicular loaded hemp lime cube specimens with the rupture stress indicated

The average stress–strain graphs for three specimens tested parallel (variant 3) and perpendicular (variant 8), with the median layer size and compaction, are shown in Fig. 3. As observed previously the failure mode exhibited when loaded parallel to the casting compaction direction was different to that when loaded perpendicular. A difficulty therefore arises in selecting a definition of compressive strength that can be compare performance in both directions. This is made harder still by the lack of a definable peak stress observed in parallel loading of cube specimens, but rather strain hardening, and a variable initial period of material settling, Fig. 3.

As insulation materials typically have much lower stiffness compared to structural materials, the compressive resistance of insulation materials, such as rigid foam, is commonly defined as the stress attained at a given strain level [24]. A similar approach has also previously been adopted in the study of bio-aggregate composites [26–28]. As the strain level that defines resistance is somewhat arbitrary, combined with the variable initial settling period for hemp-lime, an alternative approach was proposed for parallel loaded material by Tronet et al. based on a model developed for FRP-confined concretes [16]. This approach uses a repeatable method for the calculation of a yield stress that is both independent of initial displacement (settling) and compatible with prolonged strain hardening. However, whilst this approach is a development from that that of preceding studies, it is not applicable for material loaded in the perpendicular direction that do exhibit a peak stress and no strain hardening post yielding and so was not applicable to this study.

An alternative parameter is proposed in this study to compare hemp-lime material performance in both loading orientations. The composite rupture stress has been defined in this study thus: the stress at the strain where the tangent Young’s Modulus of the material, taken from a moving sample of twenty consecutive data points, falls to 25% of its peak value. While the selection of the proportion is in itself arbitrary, the threshold strain value is determined by the material’s behaviour. The proposed approach accounts for both the initial low stiffness (settling) and the different apparent stiffness of the hemp-lime in each direction, while being insensitive to post yielding behaviour.

**Fig. 4:**
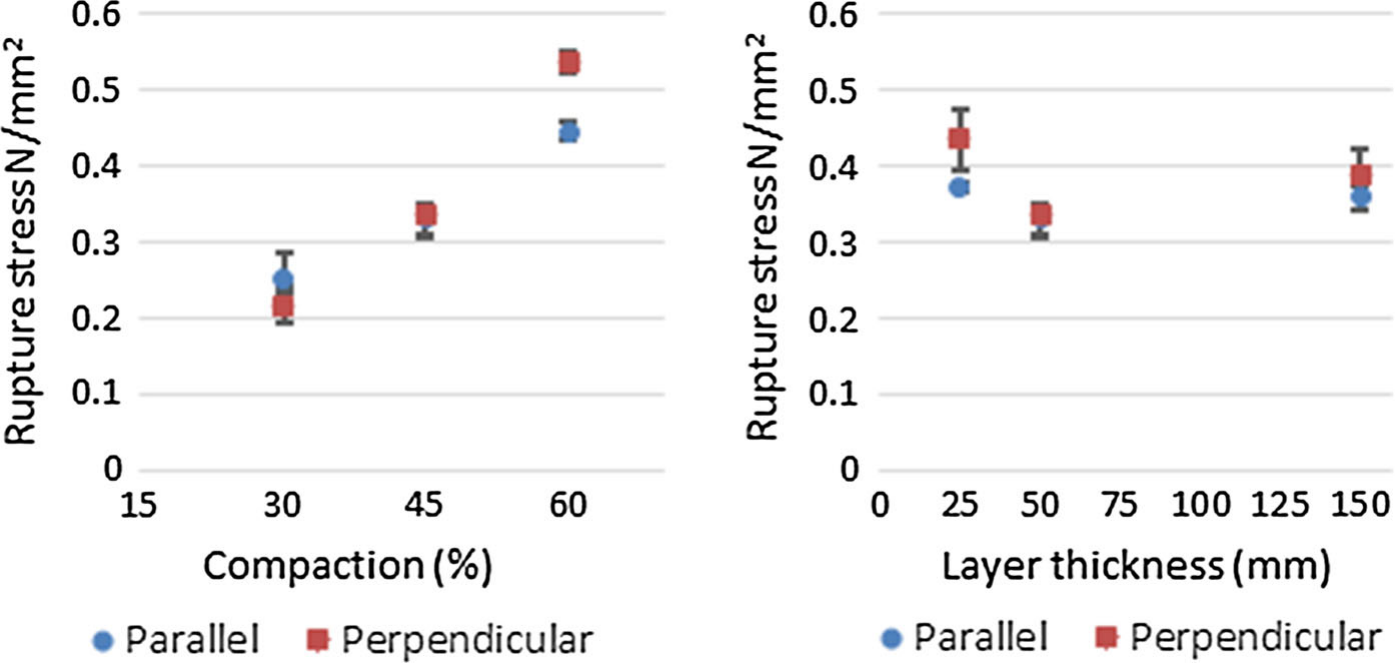
The parallel and perpendicular compressive rupture stress of hemp-lime at different compaction levels (*left*) and different layer thicknesses (*right*)

The effect of compaction and layer size on compressive rupture stress in both the parallel and perpendicular directions is shown in Fig. 4. The compressive rupture stress of hemp-lime increases with increasing casting compaction effort in both the perpendicular and parallel loaded directions, Fig. 4 (left). For a 30% increase in compaction the rupture stress increased by 200 and 175% in the perpendicular and parallel directions respectively. The average coefficient of variance from the results represented in Fig. 4 (left) was found to be 7.0%, indicating that while the natural variance in these materials is high, the increase from compaction in both cases is a significant result.

The compressive rupture stress of hemp-lime is generally independent of casting layer thickness within the range of 150to 25 mm, in both the parallel and perpendicular directions with respect to casting [Fig. 4 (right)]. There is a small apparent increase in compressive rupture strength in the perpendicular direction of 17 and 30% respectively for casting in 25 and 150 mm layers compared to 50 mm. While the average coefficient of variance for these results, 6.6%, indicates these increases may be significant, it is noted that they are of significantly lower magnitude that those observed in altering compaction, and have come from a relatively small sample size.

### Thermal conductivity

**Fig. 5:**
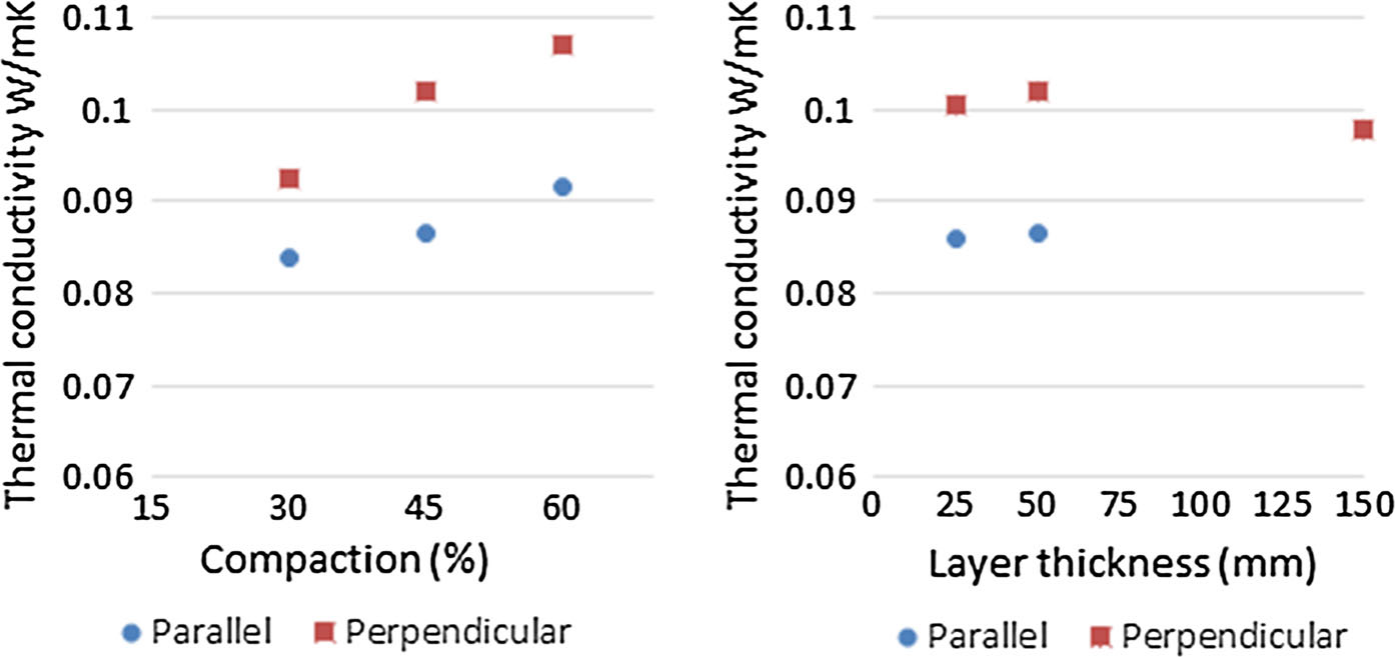
The parallel and perpendicular thermal conductivity of dry hemp-lime at different compaction levels (*left*) and different layer thicknesses (*right*)

The thermal conductivity results for the hemp-lime materials considered are presented in Fig. 5. On average the perpendicularly tested specimens exhibited a 16% higher thermal conductivity than the parallel tested specimens. While this is lower than the results reported by Pierre [20], who found a 33% directional difference in thermal conductivity, it is in line with the results of several others [29, 30]. It is observed from Fig. 5 (left) that compaction has a strong positive correlation with thermal conductivity. There is no evidence of a correlation between layer thickness and thermal conductivity in the perpendicular direction and the omission of a result for a 150 mm thick layer means no conclusive statements can be drawn in the parallel direction.

### Internal structure

**Fig. 6:**
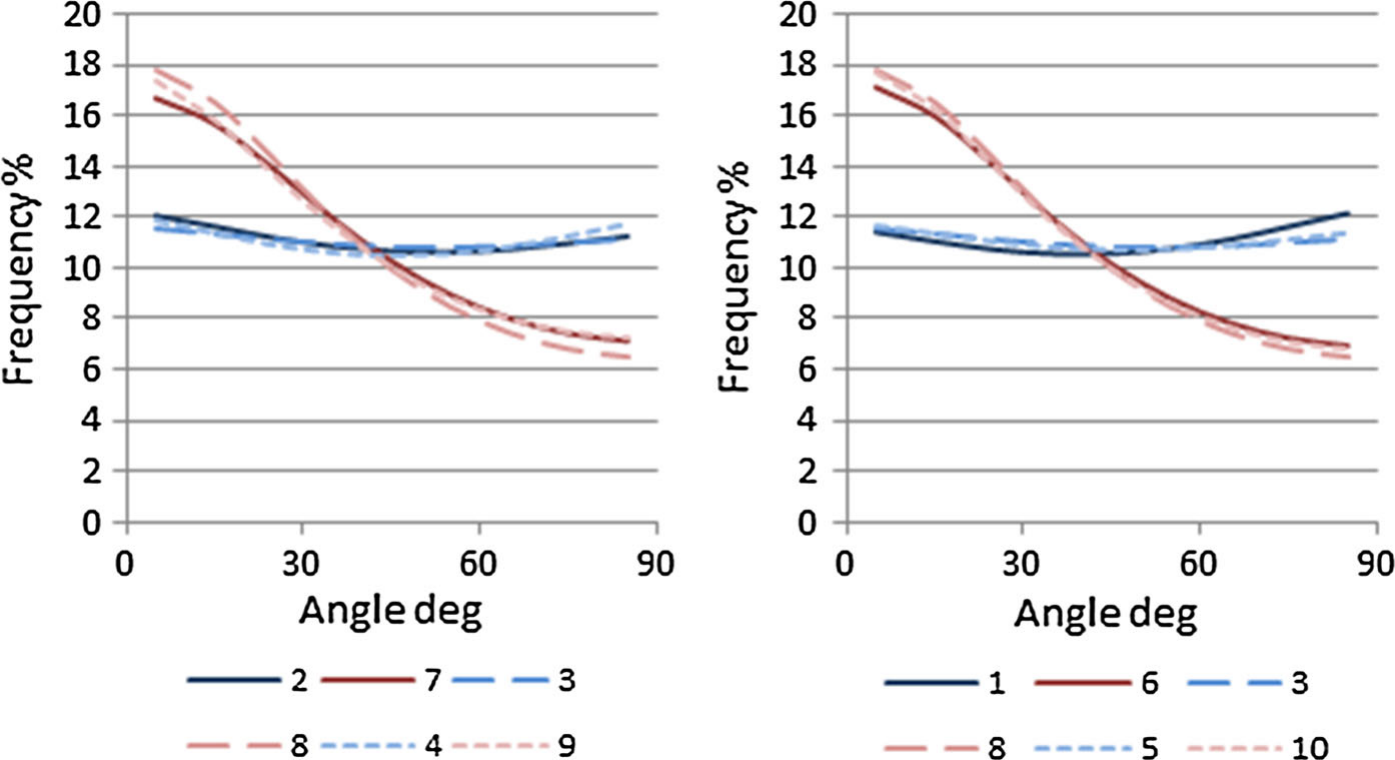
The parallel and perpendicular particle orientation frequency distributions obtained by 2D image analysis of hemp-lime at different compaction levels (*left*) and different layer thicknesses (*right*)

The results for the frequency analysis of particle orientation are presented in Fig. 6 and the volumetric proportions of air voids obtained and the 28 day density presented in Table 3. A clear difference is observed in the frequency diagrams between the perpendicular and parallel orientations, which is indicative of an anisotropic internal structure, consistent with previous results presented previously by the authors [10].

Neither compaction nor layer thickness affect the particle distribution in the parallel direction, which remained consistent in all cases (Fig. 6). The shape of the graph in this instance suggests an even and thus random distribution of particles as would be expected in planes perpendicular to the compacting force. The slightly higher frequencies at the extremes, 0° and 90°, are believed to be a result of the mould edges.

In the perpendicular direction there is again consistency in the distributions across compaction and layer thickness indicating a minimal impact. Variant 7, the lowest compaction level, appears to have the flattest graph and thus lowest degree of orientation. A relationship between compaction and degree of orientation is, however, not observed as the most compact (variant 9), is observed to have a flatter graph than the median compaction (variant 8). From Fig. 6 (right) the variant with the largest layer sizing (variant 6) appears to have a flatter graph than the similar variants of smaller layer sizes. Variations in all cases are however small and comparable to the natural variation that is assumed to be exhibited in the perpendicular distributions.

## Discussion

It is evident from the results presented in Fig. 6 that an anisotropy in the particle arrangement of the hemp in hemp-lime composites produces anisotropic thermal and mechanical behavior (Figs. 3, 5). The anisotropic arrangement of the particles is attributed to the casting process, where the application of even the lowest amount of compaction force will tend the particles into stratified planes in the perpendicular direction. As the orientation of the particles is evenly distributed in the other two axes, the result is a material with a bidirectional internal structure and physical properties.

Increasing compaction was found to produce higher compressive rupture stress and thermal conductivity in both orientations, and can be generally attributed to a reduction in the proportion of air voids in the composite, Table 3. The impact of compaction is however not global but instead is observed to have greater bearing in the perpendicular direction; a 30% increase in compaction resulting in an increase in the rupture stress of 200% in the perpendicular direction compared to 175% in the parallel directions. A differing increase of dry density for the two directions was observed, 21.7 and 18.8% respectively, put down to natural variations in the material and casting that may at least in part account for this.

**Fig. 7:**
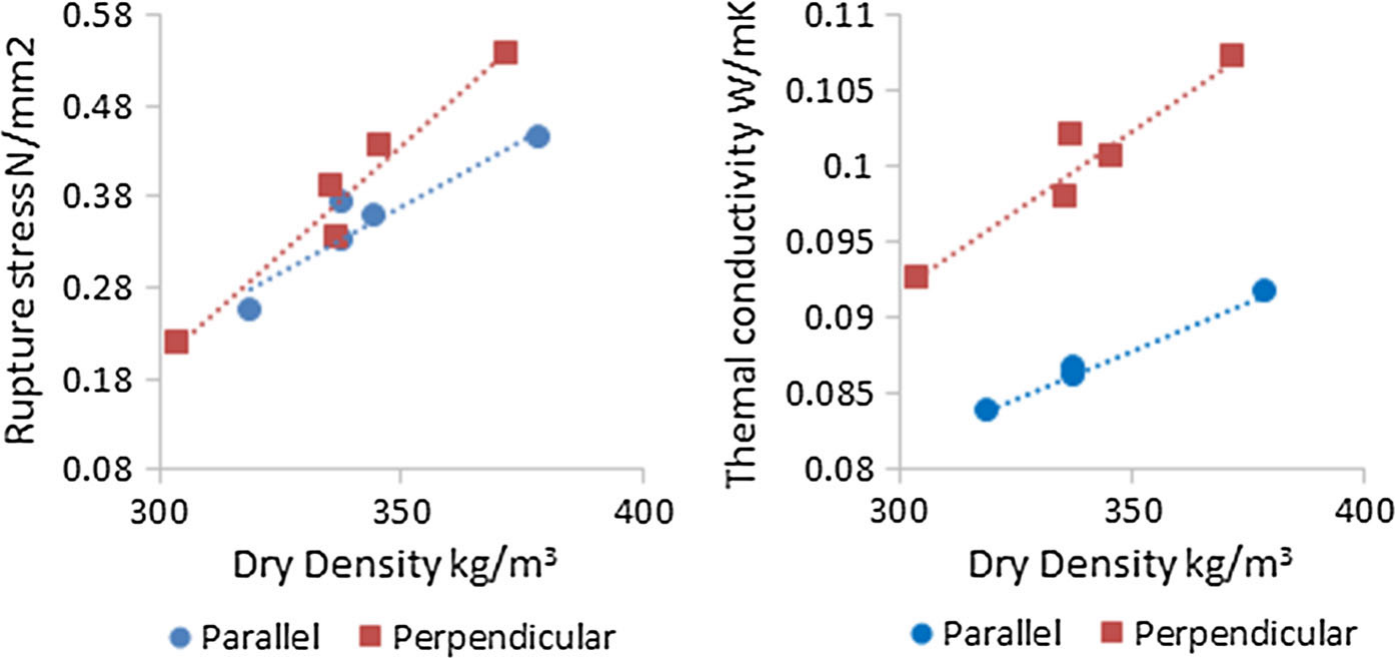
The parallel and perpendicular rupture stress (*left*) and dry thermal conductivity (*right*) with respect to density

To ascertain to what extent variation in density might explain the seemingly directionally dependent influence of compaction, the dry density can be plotted against compressive rupture stress, Fig. 7. It can be seen from Fig. 7 that the relation of dry density to compressive rupture strength is not consistent across the two directions. The differing increases of density with compaction observed in the two directions is therefore insufficient alone to account for apparent directionally dependent influence of compaction and a further explanation is needed. A logical reason would be greater compaction producing a greater degree of orientation, however, this is not supported by the image analysis that showed a lower degree of orientation present in the most compact variant. As has been found in similar analysis of other materials [31] it is possible that high compaction could increase the misidentification of touching particles, as single particles, and thus this discrepancy, may be explained by a limitation of the method used.

The layer sizes used in the casting process were not observed to influence either the thermal conductivity or the rupture stress of the material within the range of 25 to 150 mm. It had been thought that the layer size would influence the local distribution of material during casting with smaller layers leading to a more homogenous material. This in turn could reduce possible stress concentrations or thermal bridges through the material thus influencing behaviour. The results indicate that this is not the case for the range of layer sizes considered here, however, the results of others who have plotted density with depth of larger specimens indicate that it may still have bearing at larger layer sizes [8].

A common way to model the thermal conductivity and compressive strength of hemp-lime is as a linear function of dry density [12], as this is both easy to control on site and has been shown to be accurate for most cases. While a linear relationship between density and these properties is a good fit for the results obtained in this study it is also apparent that a single global linear function is not sufficient and different functions are required in the perpendicular and parallel direction respectively, Fig. 7. If it is assumed that the parallel and perpendicular functions are the bounding conditions, then a global model can be produced for thermal and physical loading in any direction by combining them into a weighted function of orientation.

To provide a form for the orientation function a comparison can be made to timber, where the compressive strength is modelled with respect to grain direction by the Hankinsons distribution [32]. As this distribution has already been found by the authors to be an appropriate form for the particle distribution within hemp-lime [10] and thus its internal structure, it is likely that it may also be appropriate for the modelling of thermal conductivity and rupture stress with respect to orientation. By substituting in the linear equations for rupture stress and thermal conductivity as found from the experimental data, Fig. 7, into the Hankinsons form, the following is derived:

1 $${\text{Rupture}}{\kern 1pt} \;{\text{stress}} = \frac{{\left( {0.0029\rho - 0.66} \right)\left( {0.0048\rho - 1.2} \right)}}{{\left( {0.0029\rho - 0.66} \right)\sin^{n} \theta + \left( {0.0048\rho - 1.2} \right)\cos^{n} \theta }},$$

2 $${\text{Thermal}}{\kern 1pt} \;{\text{conductivity}} = \frac{{\left( {0.00013\rho + 0.043} \right)\left( {0.00021\rho + 0.028} \right)}}{{\left( {0.00013\rho + 0.043} \right)\sin^{n} \theta + \left( {0.00021\rho + 0.028} \right)\cos^{n} \theta }},$$

where *ρ* is the dry density of the composite, *θ* is the direction of thermal or mechanical loading with respect to the direction of casting compaction and *n* is a constant that can be derived by fitting to experimental data. The four linear functions fitted to the data in Fig. 7 and used in Eqs. 1 and 2 have R-squared values of 0.878, 0.959, 0.996 and 0.920 respectively indicating a good level of fit of these functions and justifying there use in the model.

## Conclusions

This paper considers the influence of the casting process on the thermal and mechanical properties of hemp-lime and applies a newly developed method of image analysis to investigate the role of the internal structure in the manifestation of these properties.

A clear anisotropy in the material’s structure is observed through the image analysis for all variations considered in this study. This is attributed to the compaction produced in casting causing the elongated particles of hemp to tend towards perpendicular strata. The influence of this on the physical properties of thermal conductivity and compressive behaviour is shown to be profound and so necessitates that further study into bio-aggregate composites accounts for it.

The thickness of the layering used to cast the material was found to have no clear impact on the internal structure or the mechanical properties for the range of layer sizes considered. An initial assumption that a larger layer size would create a less homogenous material was shown not to be the case within the range of this study, however, it is considered likely that this may not apply to larger layer sizes. From a commercial standpoint it can be stated that there is no benefit to using layer sizes smaller than 150 mm during the manufacture of hemp-lime.

The degree of compaction used in casting was found to influence both the internal structure and in turn the physical properties of the composite. Increasing compaction was found to decrease the proportion of air voids in the material and thus increase both the compressive rupture stress and the dry thermal conductivity; in both cases this was found to be more pronounced in the perpendicular direction. It is considered possible that this is due to a higher level of orientation being produced at higher compaction however this was not confirmed by the image analysis possibly due to a limitation of the method.

The finding that compaction does not have a global impact on the properties of hemp-lime, but rather a directionally dependent one, is of high significance in the modelling of properties and development of the material. A new form of directionally weighted linear model for thermal conductivity and compressive strength is suggested based on a previously established model for timber, although further work is required to ascertain if the Hankinsons form of the weighting equation is more generally applicable.
